# Enhanced CO_2_ Capture Potential of Chitosan-Based Composite Beads by Adding Activated Carbon from Coffee Grounds and Crosslinking with Epichlorohydrin

**DOI:** 10.3390/ijms25168916

**Published:** 2024-08-16

**Authors:** Vorrada Loryuenyong, Worranuch Nakhlo, Praifha Srikaenkaew, Panpassa Yaidee, Achanai Buasri, Apiluck Eiad-Ua

**Affiliations:** 1 Department of Materials Science and Engineering, Faculty of Engineering and Industrial Technology, Silpakorn University, Nakhon Pathom 73000, Thailand; vorrada@gmail.com (V.L.); parn4462627@gmail.com (W.N.); srikaenkaew_p@silpakorn.edu (P.S.); yaidee_p@silpakorn.edu (P.Y.); 2 College of Materials Innovation and Technology, King Mongkut’s Institute of Technology Ladkrabang, Bangkok 10520, Thailand; apiluck.ei@kmitl.ac.th

**Keywords:** activated carbon, spent coffee grounds, biopolymer, chitosan composite materials, CO_2_ adsorption, isotherm, product innovation, waste conversion, waste recycling

## Abstract

Carbon dioxide (CO_2_) capture has been identified as a potential technology for reducing the anthropic emissions of greenhouse gases, particularly in post-combustion processes. The development of adsorbents for carbon capture and storage is expanding at a rapid rate. This article presents a novel sustainable synthesis method for the production of chitosan/activated carbon CO_2_ adsorbents. Chitosan is a biopolymer that is naturally abundant and contains amino groups (–NH_2_), which are required for the selective adsorption of CO_2_. Spent coffee grounds have been considered as a potential feedstock for the synthesis of activated coffee grounds through carbonization and chemical activation. The chitosan/activated coffee ground composite microspheres were created using the emulsion cross-linking method with epichlorohydrin. The effects of the amount of chitosan (15, 20, and 25 g), activated coffee ground (10, 20, 30, and 40%*w/w*), and epichlorohydrin (2, 3, 4, 5, 6, 7 and 8 g) were examined. The CO_2_ capture potential of the composite beads is superior to that of the neat biopolymer beads. The CO_2_ adsorbed of synthesized materials at a standard temperature and pressure is improved by increasing the quantity of activated coffee ground and epichlorohydrin. These findings suggest that the novel composite bead has the potential to be applied in CO_2_ separation applications.

## 1. Introduction

In the current and future global situation, there is an ongoing search for sustainable technologies that can effectively decrease the release of carbon dioxide (CO_2_) into the environment. The reason for the increase in the generation of greenhouse gases (GHG), which causes global warming and climate change, is primarily attributed to CO_2_ [1,2]. In 2022, it was estimated that the burning of fossil fuels emitted approximately 36.1 ± 0.3 gigatons (Gt) of CO_2_, or 70% of the total global emissions discharged into the atmosphere [3,4,5]. Therefore, it is imperative to decrease emissions and CO_2_ levels by implementing carbon capture and storage (CCS) strategies. This has become a crucial area of interest for both academic research and industry [6].

Carbon capture, utilization, and storage (CCUS) technologies are becoming more significant as they are used to facilitate the recycling of CO_2_ as a raw material. This can include replacing fossil fuels, supporting the chemical industry, or producing new carbon-based materials [7,8]. The frequently employed solid materials for CO_2_ capture include amine-based materials, alumina, mesoporous silica, metal-organic frameworks (MOFs), metal oxides, polymers, zeolites and carbon-based materials such as graphite, graphene, fullerene, carbon nanotubes, activated carbon (AC), biochar, and hydrochar [9]. Due to the acidic nature of the CO_2_ molecule, basic solid adsorbents can be generated by physically impregnating or chemically grafting a base substance onto a support surface [10]. In recent decades, there has been a rapid and significant growth in the creation of porous materials. Among these materials, the most promising ones are AC, biochar, hydrochar, and porous coordination polymers (PCP) [11].

Chitosan (CS), a polysaccharide containing an amino functionality, has been identified as a substance that can be used to create adsorbents for capturing CO_2_ [12,13,14]. CS is considered a sustainable reagent due to its natural occurrence as chitin, a significant by-product of the seafood industry [15,16,17]. It is derived from the process of N-deacetylation of chitin. CS contains a free amino group (–NH_2_) which can serve as a basic site for the adsorption of CO_2_, comparable to other adsorbents that contain amino groups [18,19,20]. Such mass-scale availability offers a chance to establish a suitable platform for CCS on demand if the issues related to the limited surface area of chitosan and, consequently, the low adsorption characteristics can be solved. This challenge, however, might be overcome by modifying CS with a suitable material with a high surface area [21,22,23].

The consumption of AC is steadily increasing in parallel with the rapid development of the economy, as it is extensively employed in a variety of applications, including air purification, hydrogen storage, gas separation, heterogeneous catalysis, and composite materials for battery electrodes and electrochromic devices (ECD) [24,25,26,27,28]. Sequentially, precursors that are inexpensive and easily accessible, including agricultural and biomass by-products or waste, have been implemented to mitigate the expense of AC [29,30]. Utilizing lignocellulosic biomass to produce valuable products like biochar or AC is one approach to establishing a sustainable and bio-based economy [31,32]. This method has the potential to decrease the accumulation of waste [33,34].

Coffee is a very significant commodity in international trade and is widely consumed as a popular beverage globally [35]. The composition of 100 kg of mature coffee cherries consisted of 39 kg of coffee pulp, 22 kg of mucilage, and 39 kg of coffee parchment [36]. The cherry is composed of several layers, including the skin, pulp, mucilage, and the protective bean shell known as the parchment. Typically, freshly harvested coffee cherries go through a series of processing procedures before they are ready to be consumed. These steps include hulling, drying, milling, polishing, and roasting [37]. On the other hand, it is essential to take into consideration the waste that is produced while one is experiencing the stimulating and motivating effects of the coffee business. Spent coffee grounds (SCGs) are a solid waste comprising 45% of the residue acquired during the brewing process. On a global scale, around 6 megatons (Mt) of SCGs are generated each year; 50% of this originates from the industrial manufacturing of instant coffee [38,39]. SCG contain a wide range of bioactive compounds, including oils, lipids, polyphenols, and polysaccharides. The great majority of these compounds have significant recycling value and are advantageous to both human health and the environment [40]. SCGs’ richness and broad variety of composition offer a significant deal of potential and versatility in making necessary products [41]. Processing is a crucial component of using SCGs. In comparison to their production, they are little used, and the procedures are inefficient. Consequently, the development of an appropriate and cost-effective process for turning these wastes into valuable materials is required [42,43].

In this study, CS was first complexed with AC from coffee grounds (CGs) and subsequently cross-linked using epichlorohydrin (EP). Typically, the composite beads (CBs) of CS are cross-linked to prevent dissolution in acidic solutions and to create microsphere morphologies [44,45,46,47,48]. CS composite materials were examined with respect to their structure and CO_2_ adsorption capabilities. The main aims of this research were to enhance the CO_2_ capture potential of CS-based CB by employing activated coffee ground (ACG) and cross-linking it with EP. An investigation was conducted to analyze the impact of varying quantities of CS, ACG, and EP. In addition, the raw materials and innovative product were analyzed using advanced techniques such as a Fourier transform infrared spectrometer (FT-IR), a thermogravimetric analyzer (TGA), an X-ray diffractometer (XRD), a particle size analyzer (PSA), a scanning electron microscope (SEM), and a surface area and porosity meter analyzer (physisorption isotherm at standard temperature and pressure (STP)). To the best of our knowledge, the utilization of these materials in the functionalization of CS/ACG microspheres is a novel contribution to the gas adsorption and separation field.

## 2. Results and Discussion

### 2.1. Characterization and Properties of CG, Carbonized Coffee Ground (CCG), and ACG

**Figure 1:**
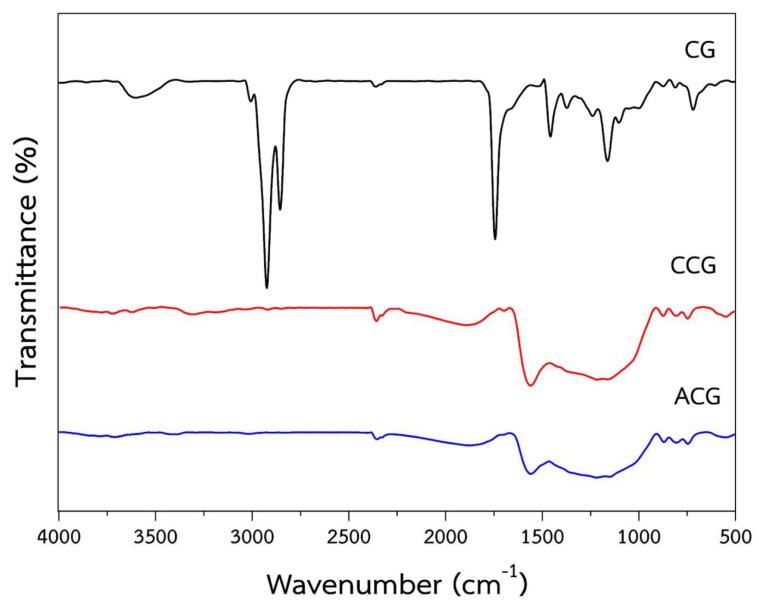
FT-IR spectra of CG, CCG, and ACG.

Organic substances like CG, which consist of lignin, hemicellulose, and cellulose, can serve as a valuable source material for producing ACG due to their exceptional adsorbent properties [49]. The CG residue is dehydrated and subsequently subjected to carbonization at a temperature of 600 °C for a duration of 1 h. This process aims to eliminate various components, including steam, volatile chemicals, and lignocellulosic substances, resulting in an increase in carbon content. Subsequently, the product will be activated in order to enhance the surface area of the CCG. The coffee residue is activated by employing chemical activation with sodium hydroxide (NaOH) in a microwave oven with a power rating of 800 W for a duration of 1 min. Figure 1 displays the FT-IR spectra of CG, CCG, and ACG. The absorption profile of CG clearly exhibited a peak at 3601 cm^−1^, which can be explained by the O–H stretching vibration [50] resulting primarily from long-chain carboxylic acids. The absorption peaks seen at 2700–2950 cm^−1^ were associated with C–H vibrations, confirming the existence of alkyl groups in CG [51]. The absorption peaks observed between 1000 cm^−1^ and 1700 cm^−1^ are attributed to the bending of C–H bonds in cellulose and hemicelluloses, as well as the stretching of C=O bonds in carboxylic acids and ester groups [52]. The range of frequencies between 600 cm^−1^ and 1000 cm^−1^ was identified as C–H bending, specifically related to aromatic ring molecules [53]. While preparing CCG and ACG, the absorption peaks in the ranges of 1000–1700 cm^−1^, 2700–2950 cm^−1^, and 3600 cm^−1^ were greatly lowered or altogether disappeared, indicating the disruption of the functional groups C=O, C–H, and O–H [54].

**Figure 2:**
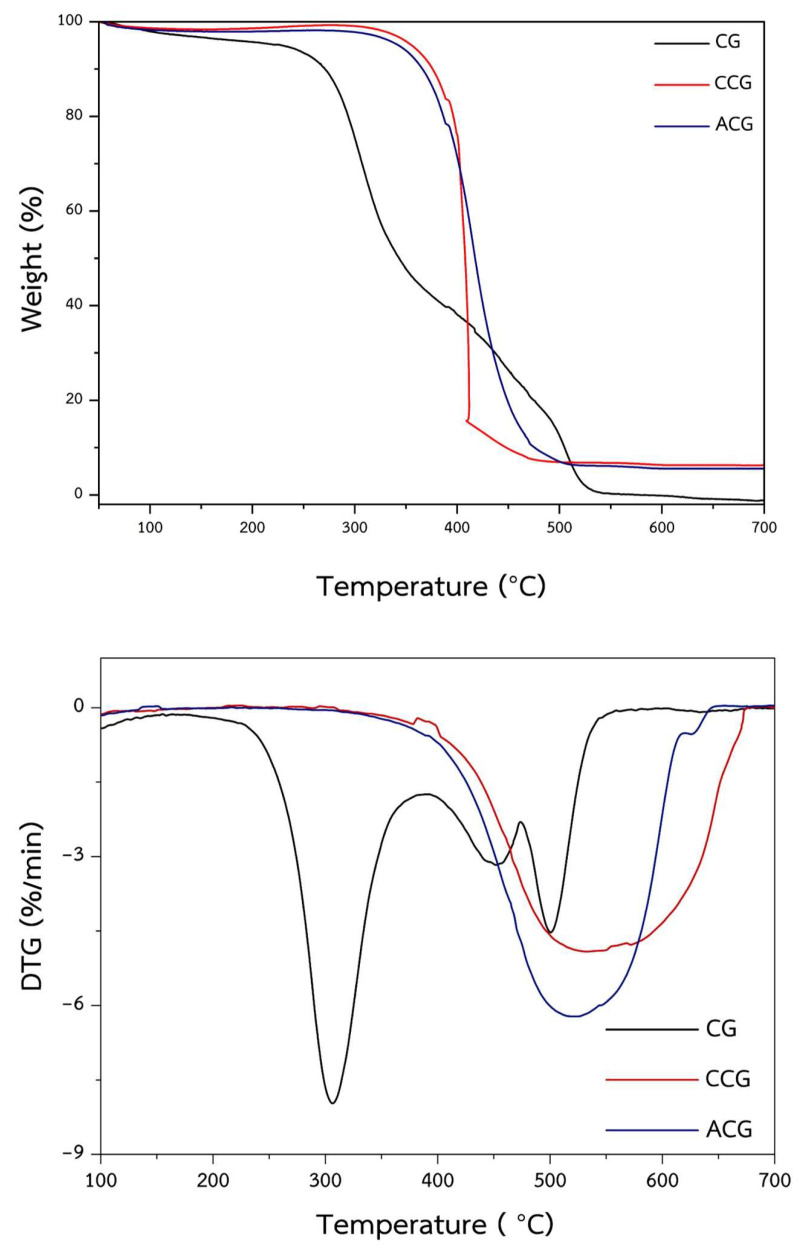
TGA and DTG thermograms of CG, CCG, and ACG.

Figure 2 depicts the four stages that are shown in the TGA and DTG thermograms of CG: In the first step, which happens below 220 °C, the mass loss (10%) is attributed to the dehydration of materials. In the second step, which occurs between 220 °C and 360 °C and corresponds to the primary carbonization, the mass loss is greater (50%) due to the elimination of the main volatile matters and tars at 300 °C. In the third step, which takes place between 360 °C and 520 °C, the mass decrease is associated with the carbonization of CG. Finally, when the temperature exceeds 520 °C, the sample is almost completely carbonized [55]. Additionally, a TGA thermogram illustrates the variation in CCG and ACG weight within a temperature range of 50 to 700 °C. The thermogram reveals a substantial reduction in mass, approximately 5%, between temperatures of 50 °C and 250 °C. The process primarily involved the elimination of moisture and potentially volatile chemicals that had accumulated within the porous structure of the material during its storage [56,57]. This outcome demonstrates the exceptional adsorption characteristics of the AC produced in this study. Subsequently, the curve exhibits a flat region extending to 350 °C, indicating the absence of a substantial mass reduction. This suggests the material possesses high thermal resistance and potential utility within this specific temperature interval. Following this particular region, there is a significant reduction in weight of around 80% between temperatures of 350 °C and 700 °C. The reduction in mass is caused by the breakdown of the oxygenated groups and the partial deterioration of the carbon structure [58,59].

**Figure 3:**
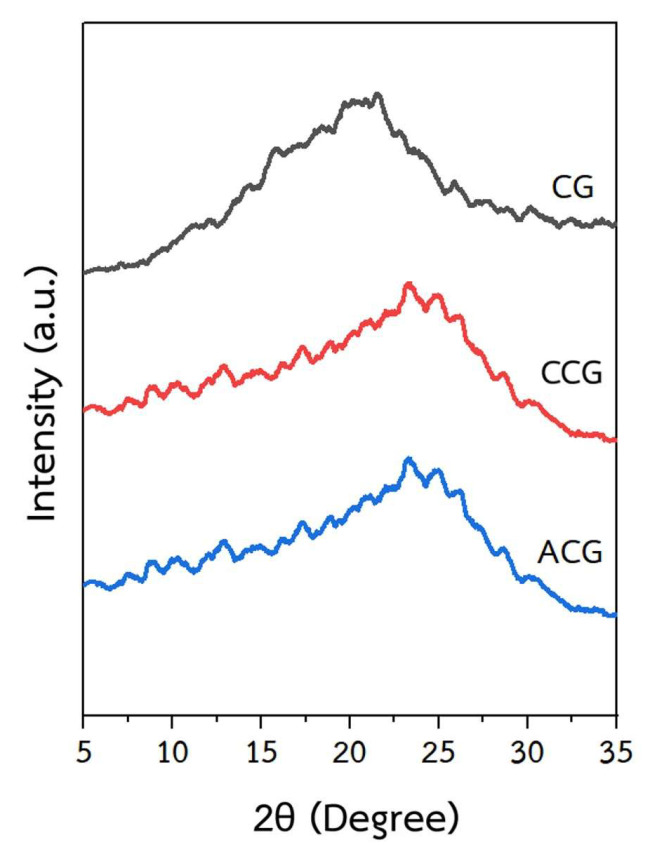
XRD patterns of CG, CCG, and ACG.

XRD is a commonly employed method for analyzing the structure of materials in the bulk or powdered state. The powder XRD patterns show significant intensity in the low-angle scattering region (Figure 3). All materials exhibited a comparable XRD pattern, which additionally indicated the presence of pores in both the CCG and ACG. Moreover, the diffraction peak observed at approximately 23° of 2-theta corresponds to the diffraction plane (0 0 2) of amorphous carbonaceous materials, as reported in references [60,61,62]. Nevertheless, it is evident that the diffraction peaks exhibit substantial broadening, indicating that the structure of the produced ACG is composed of small crystalline phases scattered among extensive disordered regions [57,63].

**Figure 4:**
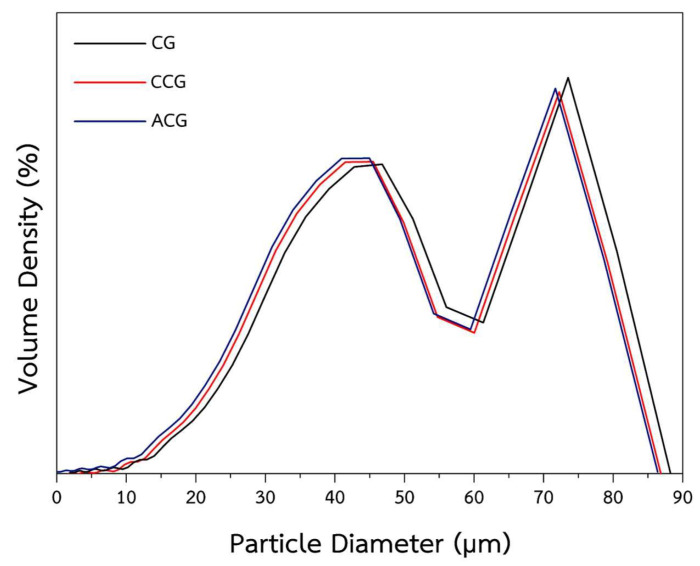
Particle size analysis of CG, CCG, and ACG.

The particle size distribution of the CG, CCG, and ACG ranges from 10 to 90 μm, as represented in Figure 4. The CG particles were determined to have sizes within the two ranges 10–60 μm and 60–85 μm. Upon comparing the particles produced from carbonization and chemical activation, which were thought to be closer to natural raw materials, it was seen that the shapes and sizes of CCG were comparable to CG [64]. Additionally, the biochar degraded along the same areas of vulnerability. The ACG particles in the two size classes had a similar real size to that of CG and CCG, but were slightly smaller than CG and CCG [65,66].

**Figure 5:**
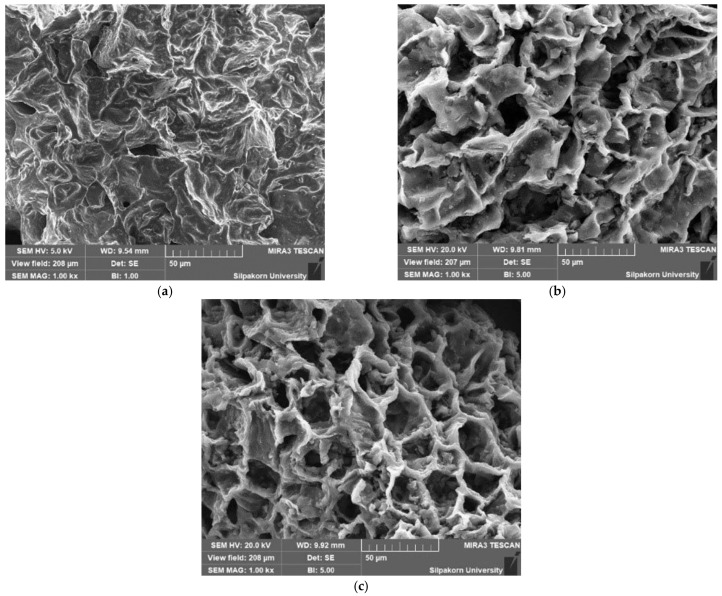
SEM images at 1000× magnification of (**a**) CG, (**b**) CCG, and (**c**) ACG.

A SEM was utilized to examine the structure and dimensions of materials. Figure 5 displays SEM micrographs of CG, CCG, and ACG samples magnified 1000 times. The surface of the CG showed a smooth texture, with the presence of oil permeating the pores. The morphology closely resembles the findings of another study [67,68], where the observed pores function as pathways for the microporous network. The microscopic examinations revealed distinct variations in the textural characteristics of the CCG and ACG samples compared to the CG sample. The images demonstrated the degree of irregularities on the surfaces of CCG and ACG, which exhibited a greater level of roughness, a highly porous structure, and numerous voids when compared to the raw material from SCG [69].

The porous structures of carbon materials have a substantial impact on their ability to adsorb CO_2_. Table 1 displays the specific surface area, pore size, and pore volume of CG, CCG, and ACG. In comparison to the textural characteristics, the Brunauer–Emmett–Teller (BET) specific surface area of these materials exhibited a considerable increase, with the order being ACG (5.60 m^2^/g) > CCG (4.39 m^2^/g) > CG (2.90 m^2^/g). A comparable pattern was also noted in the pore volume findings [70,71]. Hence, the permeable arrangement of the carbon components is directly related to the process of carbonization and chemical activation.

**Figure 6:**
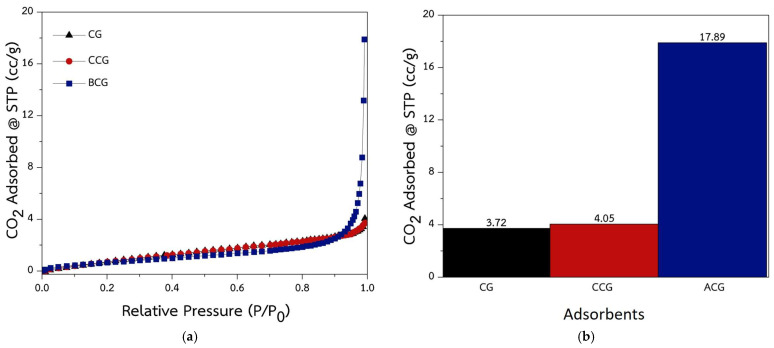
Adsorption isotherms of CO_2_ by CG, CCG, and ACG at relative pressure (**a**) from 0 to 1, and (**b**) 1.

Figure 6 presents the CO_2_ adsorption isotherms for CG, CCG, and ACG across a range of relative pressures (P/P_0_) from 0 to 1. The adsorption isotherm of adsorbents for CO_2_ is mainly characterized as type I [72]. The isotherms of ACG exhibit the distinctive features of materials with a high degree of microporosity, where almost all of the CO_2_ adsorption occurs at a relative pressure of 0.9. This is followed by a sudden change in the knee and plateau [73]. The CO_2_ capture potential of ACG is significantly higher at 17.89 cc/g compared to CCG at 4.05 cc/g and CG at 3.72 cc/g. The observed phenomena `agree with the SEM images presented in Figure 5. Additionally, the associated textural characteristics of adsorbents are clearly explained in Table 1. Adsorbent ACG has the greatest pore volume, measuring approximately 0.87 cm^3^/g, which is greater than the pore volumes of CCG (0.62 cm^3^/g) and CG (0.57 cm^3^/g)

### 2.2. Characterization and Properties of CS/ACG Composite Materials

**Figure 7:**
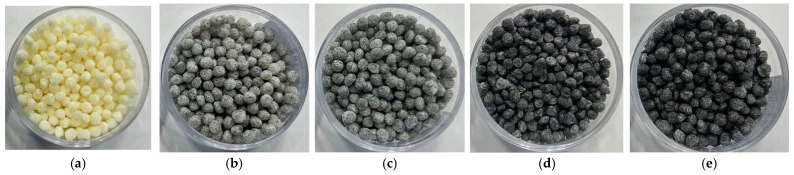
Digital images of (**a**) CB_20/0%_, (**b**) CB_20/10%_, (**c**) CB_20/20%_, (**d**) CB_20/30%_, and (**e**) CB_20/40%_.

Figure 7 shows the digital photographs of the prepared CS beads and CS/ACG beads. The wet adsorbent beads exhibited a soft and spherical morphology. The CBs underwent a process of hardening and lost approximately 50–55% of their water content after drying until a consistent mass was achieved, while still maintaining their original shape [74]. From the images, it is clear that the beads were perfectly round and had a diameter of around 2.4 mm. The CS beads without any particles of ACG (CB_20/0%_) displayed a pearl white color, as shown in Figure 7a. Upon the addition of ACG in CS, the CS/ACG beads experienced a color change, turning gray (Figure 7b), and eventually dark gray when the ACG content exceeded 20%*w/w* (Figure 7d,e). Furthermore, the dried CS beads had an average surface area of 2.33 m^2^/g. A higher surface area of the CS/ACG beads could result in a greater degree of CO_2_ adsorption [75].

**Figure 8:**
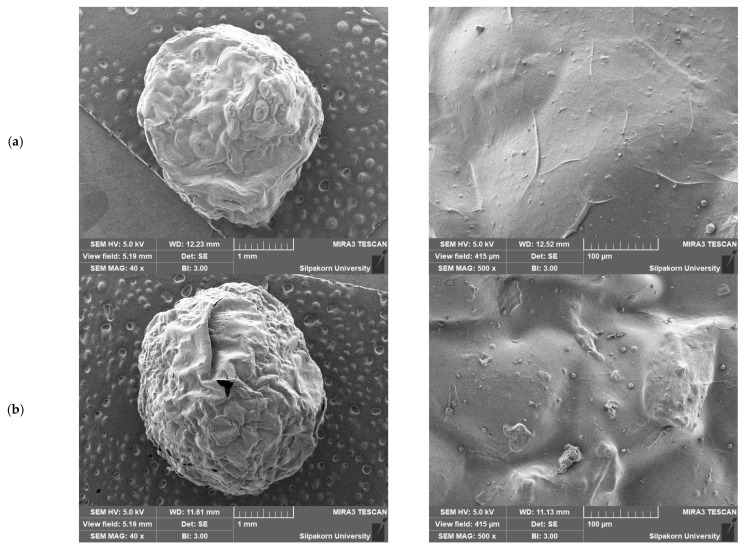

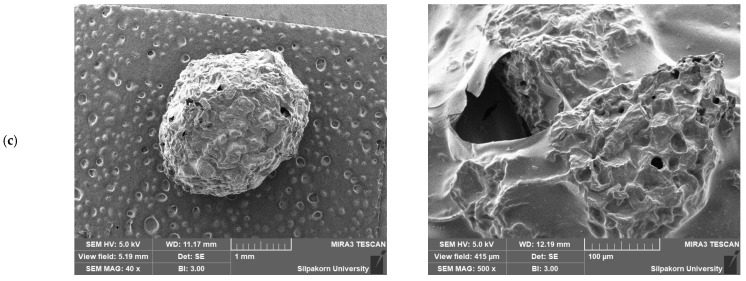
SEM images at 40× and 500× magnification of (**a**) CB_20/0%_, (**b**) CB_20/20%_, and (**c**) CB_20/40%_.

Figure 8 demonstrates the SEM images of both CS beads and CS/ACG beads. The microspheres possess a symmetrical spherical configuration. The micro pore structures are observable on the surface and interface of the microspheres. The porous network structure serves two purposes: firstly, it effectively prevents the loss of ACG particles to a certain degree, and secondly, it enhances the diffusion of gaseous substances and provides a greater contact surface to assist CO_2_ adsorption [76]. When comparing CS beads with CS/ACG beads, the amount of ACG in CS/ACG beads increased from 0%*w/w* to 20%*w/w* and 40%*w/w*, respectively. The CB_20/0%_ exhibit a consistent and homogeneous distribution, as depicted in Figure 8a. The surface morphology of CS beads reveals macropore formations, wherein the pores are interconnected [77]. The inclusion of ACG results in the microspheres exhibiting increased microporous and mesoporous structures of CB_20/20%_, and CB_20/40%_, as well as a rougher surface (Figure 8b,c).

**Figure 9:**
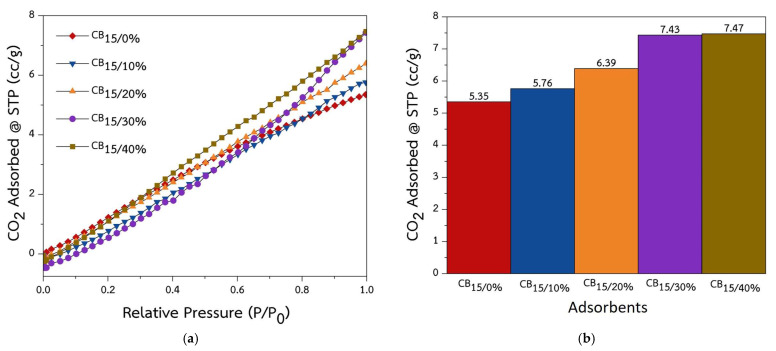
Adsorption isotherms of CO_2_ by CB_15/0%_, CB_15/10%_, CB_15/20%_, CB_15/30%_, and CB_15/40%_ at relative pressure (**a**) from 0 to 1, and (**b**) 1.

**Figure 10:**
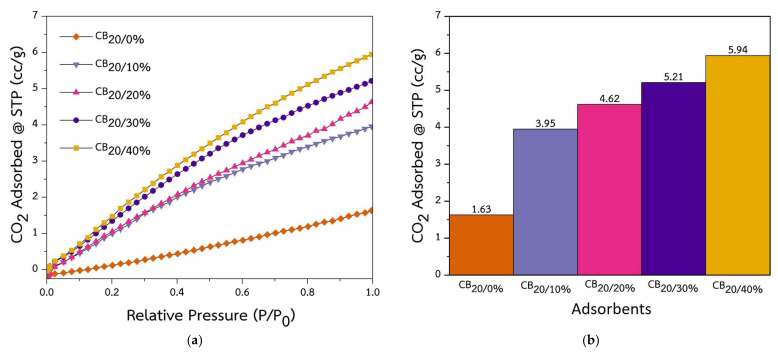
Adsorption isotherms of CO_2_ by CB_20/0%_, CB_20/10%_, CB_20/20%_, CB_20/30%_, and CB_20/40%_ at relative pressure (**a**) from 0 to 1, and (**b**) 1.

**Figure 11:**
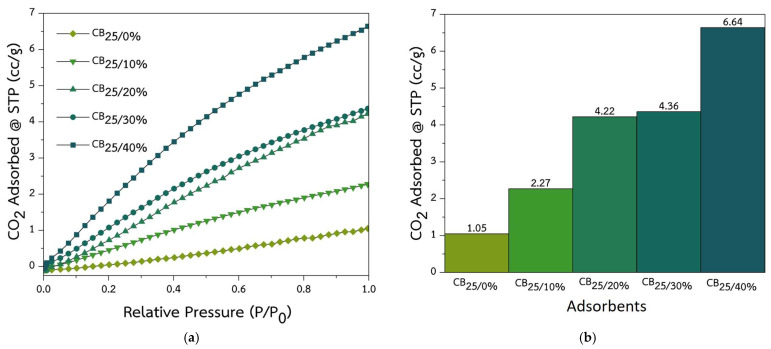
Adsorption isotherms of CO_2_ by CB_25/0%_, CB_25/10%_, CB_25/20%_, CB_25/30%_, and CB_25/40%_ at relative pressure (**a**) from 0 to 1, and (**b**) 1.

**Figure 12:**
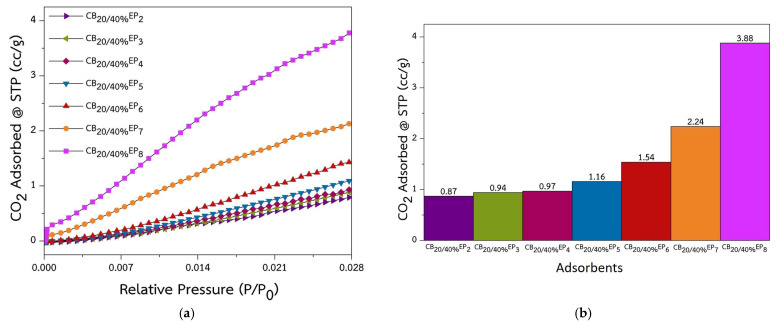
Adsorption isotherms of CO_2_ by CB_20/40%_EP_2_, CB_20/40%_EP_3_, CB_20/40%_EP_4_, CB_20/40%_EP_5_, CB_20/40%_EP_6_, CB_20/40%_EP_7_, and CB_20/40%_EP_8_ at relative pressure (**a**) from 0 to 1, and (**b**) 1.

In order to examine how input parameters impact the CO_2_ adsorption capabilities of the CS/ACG composite, a perturbation plot was developed and is displayed in Figure 9, Figure 10, Figure 11 and Figure 12. The adsorbents’ capacity for capturing CO_2_ is investigated throughout numerous parameters, such as varying quantities of CS (15–25 g), ACG content (10–40%*w/w*), EP amount (2–8 g), and relative pressure (0–1). Regarding the ACS/CS mass ratio, the sample with a larger weight of ACS demonstrates an enhancement in the composite’s ability to adsorb CO_2_, resulting in a higher amount of CO_2_ being adsorbed at a STP. The increased ACS component in the composite leads to a greater specific surface area, pore size, and pore volume of the CB. However, it also results in decreased density and enhanced total adsorption capacity compared to pure samples like CS bio adsorbent [78]. The CB_15_ has a larger ACS/CS mass ratio compared to CB_20_ and CB_25_. Hence, the sequence of CO_2_ absorption is as follows: CB_15_ has the highest intake, followed by CB_20_, and then CB_25_.

**Scheme 1:**
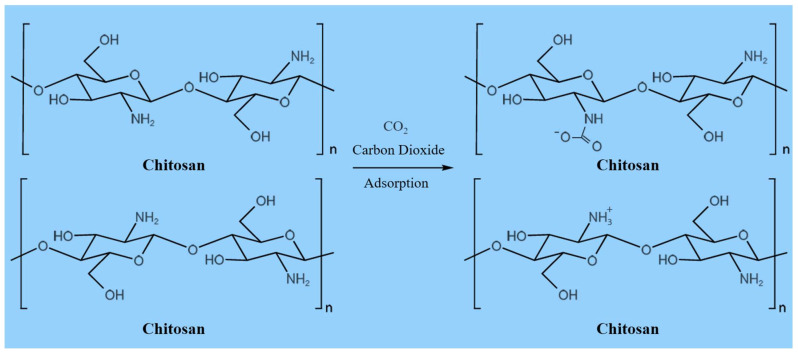
Adsorption of CO_2_ by CS.

Moreover, the CS and ACG samples have a high concentration of nitrogen and oxygen, respectively. They also contain several functional groups such as –NH_2_, –COOH, –NO_2_, and –OH that withdraw the electrons of other molecules [79,80]. These characteristics make the CS/ACG sample a highly promising option for applications involving the adsorption of CO_2_ (Scheme 1). The dispersion of the mentioned functional group on the surface of the CB sample leads to increased heterogeneity of the composite sample. This, in turn, enhances the ability of the CS/ACG composite to adsorb CO_2_ by improving the dipole–quadrupole interaction between the surface of the adsorbent and CO_2_ molecules [81].

**Scheme 2:**
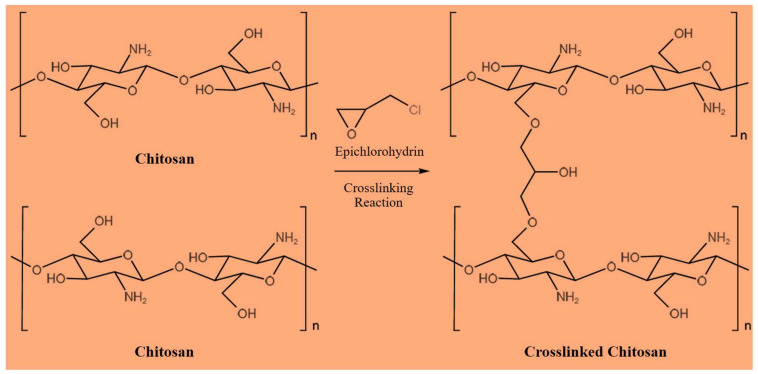
Crosslinking of CS with EP.

CS is commonly crosslinked to prevent dissolution in acidic conditions and to produce microspheres. It was combined with ACG powder and subsequently cross-linked with EP. The cross-linker will chemically react with the amino groups or hydroxyl groups of CS, resulting in an enhancement of CS’s acid stability and adsorption capacities (Scheme 2). When EP was reacted with –NH_2_ or –OH groups of CS, it resulted in the cross-linking of CS chains, leading to the formation of a hard microsphere after cross-linking. The CO_2_ adsorption efficiency of CS/ACG beads was examined through changing the EP crosslinking amounts, as depicted in Figure 12. As the concentration of the crosslinking agent added to the CB increased, the capacity for adsorbing CO_2_ similarly increased in a sequential manner. The increased porosity and surface area of the CB resulting from the crosslinking process allows for greater accessibility of carbon dioxide molecules [82]. In addition, crosslinking enhances the acid/basic resistance of CS, enabling it to preserve its adsorption capabilities throughout a broader spectrum of circumstances. Increasing the content of the crosslinking agent enhances the efficiency of CO_2_ adsorption.

**Figure 13:**
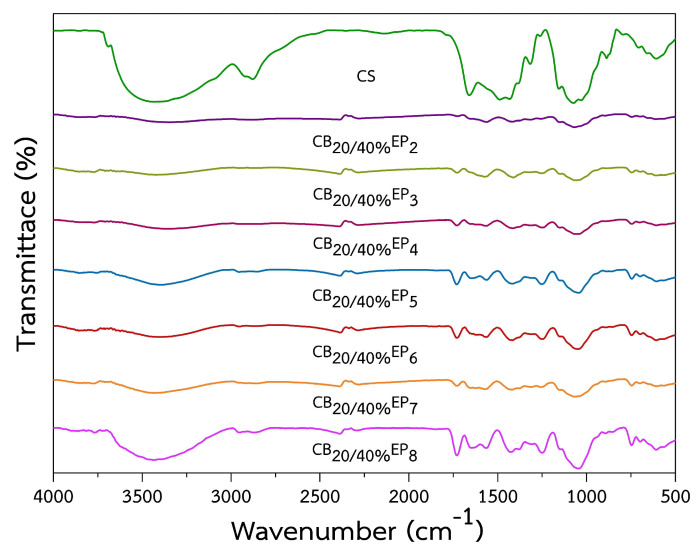
FT-IR spectra of CS, CB_20/40%_EP_2_, CB_20/40%_EP_3_, CB_20/40%_EP_4_, CB_20/40%_EP_5_, CB_20/40%_EP_6_, CB_20/40%_EP_7_, and CB_20/40%_EP_8_.

Figure 13 displays the FT-IR spectra of pure CS. The infrared spectrum exhibits distinct peaks at specific wavenumbers: 3440 cm^−1^ (resulting from the stretching of −OH and N−H groups, which overlap), 2870 cm^−1^ (associated with the stretching of C−H bonds), 1660 cm^−1^ (corresponding to Amide I), 1595 cm^−1^ (representing the amide II band (N−H)), 1325 cm^−1^ (related to Amide III), 1090 cm^−1^ (attributed to the stretching of C−O bonds in secondary hydroxyl groups), 1035 cm^−1^ (associated with the stretching of C−O bonds in primary hydroxyl groups), and an absorption band at 895 cm^−1^ caused by the β-(1,4) glycosidic bond in CS. Previous studies [83,84] have revealed the same findings for the FT-IR spectrum of the CS bead. When the EP concentration in the CB was increased, the peak of the −OH functional group and −NH_2_ functional group underwent changes. The disappearance of the −NH_2_ peak observed with increased EP content suggests a reduction in amine groups resulting from the crosslinking of CS chains by EP. Nevertheless, the −OH peak exhibited broadening as the EP concentration increased, suggesting an augmentation in hydroxy groups resulting from the higher EP content. This indicates the presence of a crosslinked structure in CS/ACG combining carbon atoms, oxygen atoms, and hydrogen atoms of EP. Furthermore, the intensity of the C−O functional group (1045 cm^−1^) increased as the EP content increased, indicating the creation of new C−O bonds between CS and EP during the process of crosslinking.

The values of CO_2_ adsorption on CS polymeric materials that have been obtained by other research groups are summarized in Table 2. The CO_2_ capture potential of the CS-based CB from this research is consistent with the range of values reported by other researchers [10,19,85,86].

## 3. Experimental

### 3.1. Materials and Chemicals

**Scheme 3:**
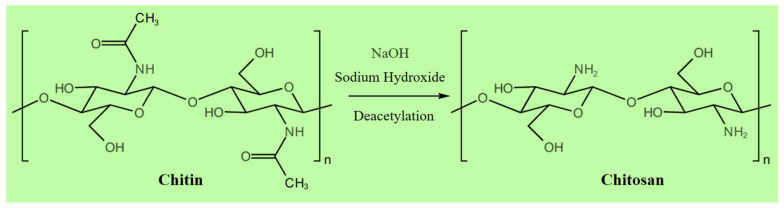
Deacetylation of chitin with NaOH.

The CS powder, with a particle size of 200 mesh and a degree of deacetylation greater than 90%, was supplied by Sinudom Agriculture Products located in Surat Thani province, Thailand. The N-deacetylation of chitin is the chemical process that results in the formation of CS (Scheme 3). The SCG was obtained from Café Amazon, a coffee business situated in Silpakorn University, Nakhon Pathom, Thailand. The EP and other chemicals used in the study were of analytical grade, purchased from Merck Ltd., Bangkok, Thailand, with a purity level exceeding 99%. These chemicals were used without any modifications.

### 3.2. Preparation of ACG

The CG was rinsed with distilled water, filtered, and dried in an oven at 80 °C for 24 h. Subsequently, the CG was crushed to achieve a particle size less than 150 µm (100 mesh). Afterwards, the CG material was subjected to carbonization at a temperature of 600 °C for a duration of 1 h. The heating rate was set at 10 °C per minute, and the process was carried out under a flow of nitrogen gas (N_2_). The CCG was immersed in a KOH solution for a duration of 24 h, with a CCG to KOH ratio of 0.75:1 by weight. Afterward, the soaked substance underwent chemical activation in a microwave oven, utilizing an electrical power of 800 watts for a duration of 1 min. The collected ACG was cleaned by filtration and rinsed with distilled water until it reached a pH of around 7.0. Finally, it was dried in an oven at a temperature of 100 °C for a duration of 24 h.

### 3.3. Synthesis of CS/ACG Composite Microsphere

**Scheme 4:**
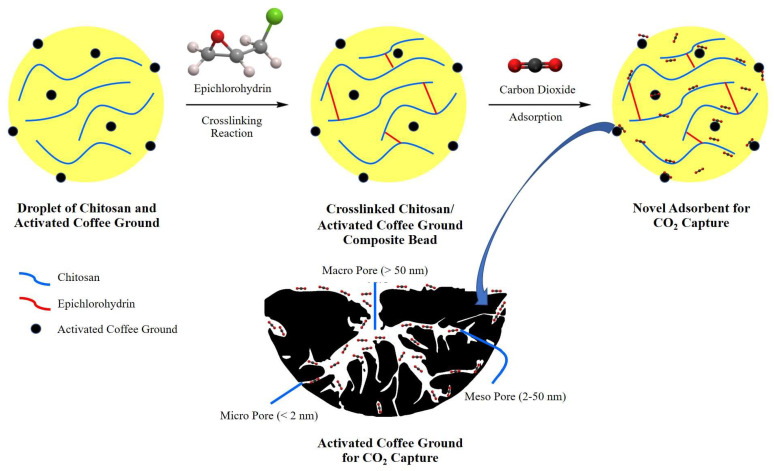
Preparation of novel CO_2_ adsorbent from CS, ACG and EP.

The CS was initially added to a 0.5 M acetic acid solution with a volume of 500 mL. The quantities of CS added were 15, 20, and 25 g, and the mixture was agitated for 5 h. ACG was subsequently included into various CS solutions in varying quantities, ranging from 0 to 40%*w/w* of CS. The mixtures were then thoroughly blended by stirring them for 30 min, which produced homogenous CS and ACG combinations. Afterwards, the slurry was cautiously introduced into a beaker containing 6 %v/v 1000 mL ammonia solution using a syringe. The creation of spherical beads was initiated by this procedure, followed by numerous washes with distilled water to eliminate contaminants. The cleaned sample was thereafter immersed in a solution containing 2–8 g of EP dissolved in 50 mL of ethanol. The presence of this suspension allowed for the formation of strong connections between the CS and EP, resulting in improved durability and stability. In order to finalize the preparation of the novel CS/ACG composite microsphere (Scheme 4), the materials were subjected to a comprehensive freeze-drying process at a temperature of −54 °C for a duration of 24 h. The different amounts of CS, ACG, and EP required for the synthesis of CS/ACG composite microspheres in this investigation are presented in Table 3 and Table 4.

### 3.4. Material Characterization

The characterization of the materials’ functional groups was conducted using an FT-IR Spectrometer (VERTEX 70v, BRUKER, Leipzig, Germany). The sample was ground with potassium bromide (KBr) and compressed into a pellet. It was then analyzed utilizing a wave number range of 4000–500 cm^−1^. The thermal stability of the particle was examined using a TGA/DSC 1 Star System (Mettler-Toledo GmbH, Giessen, Germany). The material samples were heated to 700 °C at a rate of 5 °C/min in the presence of a N_2_ flow. The XRD patterns were obtained using a LabX XRD-6100 instrument (Shimadzu, Kyoto City, Japan) equipped with Cu-Kα radiation, operating at 30 kV and 20 mA. The measurements were taken in the 2θ range of 5° to 35°, with a step size of 0.04°. The powder’s particle size distribution was determined using a Beckman Coulter LS 100 Q Laser Diffraction PSA (NC, USA). The SEM (MIRA3, TESCAN, Brno—Kohoutovice, Czech Republic) was used to analyze the morphologies and microstructures of the materials. All of the samples were subjected to degassing at a temperature of 150 °C for a duration of 2 h under vacuum conditions. Physisorption-isotherms were then obtained using a surface area and porosity meter analyzer. The adsorbent samples’ surface area and porosity were analyzed using sorption isotherms obtained at a temperature of −196 °C on the Autosorb IQ-MP (3 STAT) instrument manufactured by Anton Paar Germany GmbH (Ostfildern-Scharnhausen, Germany). The adsorption data were tested using a relative pressure range of 0.001 to 0.999. Prior to the analyses, the materials were subjected to degassing at a temperature of 120 °C for a duration of 24 h. The surface area was determined using the BET model, whereas the pore volume was acquired by the Barrett−Joyner−Halenda (BJH) approach.

## 4. Conclusions

Within the context of the circular economy, the SCGs were transformed into powdered ACs through pyrolysis at a temperature of 600 °C for a duration of 1 h, under a N_2_ atmosphere. The ACs were then chemically activated using KOH as the activating agent, in a microwave oven with a power output of 800 W, for a duration of 1 min. A comparison was made between the textural characteristics and CO_2_ adsorption effectiveness of CG, CCG, and ACG. The ranking of the particle surface area is as follows: ACG (5.60 m^2^/g) > CCG (4.39 m^2^/g) > CG (2.90 m^2^/g). Subsequently, composite microspheres consisting of CS and ACG were synthesized using the emulsion cross-linking technique, and their capacity to adsorb CO_2_ was investigated. The adsorption of CO_2_ is contingent upon the quantity of ACG, CS, and crosslinking agent employed during the fabrication of composite materials. Increasing the amount of ACG and EP enhances the efficacy of CO_2_ adsorption in materials. This discovery also provides a technique for producing innovative porous polymers with a substantial capacity for adsorbing CO_2_. Thus, the CB shows great potential for use in eliminating CO_2_ emissions from industrial facilities.

## Figures and Tables

**Table 1:** Textural properties of CG, CCG, and ACG.

Sample	Surface Area (m^2^/g)	Pore Size (nm)	Pore Volume (cm^3^/g)
CG	2.90	3.31	0.57
CCG	4.39	3.94	0.62
ACG	5.60	3.71	0.87

**Table 2:** CO_2_ adsorption on CS polymeric materials.

Sample	CO_2_ Adsorption (mmol/g)	Reference
Chitosan/Activated Coffee Ground	0.34	This Work
Imine-Chitosan	0.11	[85]
Triphenyl Amine Porous Chitosan Derivative	0.85	[86]
Chitosan-Hydrothermal Carbonization	0.30	[10]
Chitosan/Fumed Silica	0.29	[19]

**Table 3:** The amount of CS, ACG, and EP required for CB synthesis using a constant amount of EP.

Sample	CS (g)	ACG (g)	ACG (%*w*/*w*)	EP (g)
CB_15/0%_	15	0	0	5
CB_15/10%_	15	1.5	10	5
CB_15/15%_	15	3.0	20	5
CB_15/20%_	15	4.5	30	5
CB_15/25%_	15	6.0	40	5
CB_20/0%_	20	0	0	5
CB_20/10%_	20	2.0	10	5
CB_20/20%_	20	4.0	20	5
CB_20/30%_	20	6.0	30	5
CB_20/40%_	20	8.0	40	5
CB_25/0%_	25	0	0	5
CB_25/10%_	25	2.5	10	5
CB_25/20%_	25	5.0	20	5
CB_25/30%_	25	7.5	30	5
CB_25/40%_	25	10.0	40	5

**Table 4:** The amount of CS, ACG, and EP required for CB synthesis with different amounts of EP.

Sample	CS (g)	ACG (g)	ACG (%*w/w*)	EP (g)
CB_20/40%_EP2	20	8.0	40	2
CB_20/40%_EP3	20	8.0	40	3
CB_20/40%_EP4	20	8.0	40	4
CB_20/40%_EP5	20	8.0	40	5
CB_20/40%_EP6	20	8.0	40	6
CB_20/40%_EP7	20	8.0	40	7
CB_20/40%_EP8	20	8.0	40	8

## Data Availability

All the data are available from the corresponding author, Achanai Buasri, email: achanai130@gmail.com.

## Abbreviations

AC, activated carbon; ACG, activated coffee ground; BJH, Barrett-Joyner-Halenda; BET, Brunauer-Emmett-Teller; CCG, carbonized coffee ground; CB, composite bead; CCS, carbon capture and storage; CCUS, carbon capture, utilization, and storage; CG, coffee ground; CS, chitosan; ECD, electrochromic device; EP, epichlorohydrin; FT-IR, Fourier transform infrared spectrometer; GHG, greenhouse gas; MOF, metal-organic framework; PSA, particle size analyzer; PCP, porous coordination polymers; SEM, scanning electron microscope; SCG, spent coffee ground; STP, standard temperature and pressure; TGA, thermogravimetric analyzer; XRD, X-ray diffractometer.
